# Extracorporeal cardiopulmonary resuscitation for adult patients who underwent post-cardiac surgery

**DOI:** 10.1186/s40001-015-0179-4

**Published:** 2015-10-12

**Authors:** Yanyan Zhao, Jialin Xing, Zhongtao Du, Feng Liu, Ming Jia, Xiaotong Hou

**Affiliations:** Department of Extracorporeal Circulation, Center for Cardiac Intensive Care, Beijing Anzhen Hospital, Beijing Institute of Heart Lung and Blood Vessel Diseases, Capital Medical University, Beijing, 100029 China

**Keywords:** Refractory cardiac arrest, Extracorporeal cardiopulmonary resuscitation, Cardiac surgery, Mean extracorporeal membrane oxygenation

## Abstract

**Background:**

Refractory cardiac arrest (CA) occasionally develops in patients after cardiac surgery.

**Objective:**

To examine the clinical outcomes of extracorporeal cardiopulmonary resuscitation (ECPR) in adult patients with post-cardiotomy CA.

**Methods:**

This was a retrospective study of the 9-year experience (from January 2004 to May 2012) of the Beijing Anzhen Hospital with ECPR in adult patients with post-cardiotomy CA. At this hospital, a dedicated ECPR team is available 24/7 for emergency cases requiring ECPR. Demographic data, biochemical data, survival, morbidity, and complications were examined before, during, and after ECPR. Outcomes were compared between survivors and non-survivors.

**Results:**

Twenty-four adult patients (19 men and 5 women; mean age: 59.3 ± 11.9 years) received ECPR support for post-cardiotomy CA. The cardiac surgery procedures included coronary artery bypass grafting (*n* = 20, 83.3 %), valvular surgery alone (*n* = 2, 8.3 %), and correction of congenital heart defects (*n* = 2, 8.3 %). The mean extracorporeal membrane oxygenation (ECMO) duration was 115.23 ± 70.17 h. Twenty-one patients received ECPR after intra-aortic balloon pump, and three patients received ECPR directly. The main cause of mortality was multiple system organ failure (*n* = 12, 50.0 %). Approximately one-half of non-survivors had severe neurologic impairments. Among 16 patients who were weaned off ECMO support, eight patients survived to hospital discharge.

**Conclusions:**

ECPR can be effective for partial cardiopulmonary support to resuscitate adult patients suffering from refractory CA after cardiac surgery. Improvement in outcomes of patients who received ECPR requires a multidisciplinary approach to protect organ function and limit organ injury before and during cardiac support.

## Background

Cardiac arrest (CA) is a major health concern, and survival rates remain very low despite early access to emergency medical care and continual improvements in treatment strategies [1]. The incidence of myocardial dysfunction after cardiac surgery is 3–5 %, and most patients can be managed using inotropes or intra-aortic balloon counterpulsation [2]. Nevertheless, about 1 % of these patients will experience refractory cardiac dysfunction and will need advanced mechanical support [3, 4].

Extracorporeal cardiopulmonary resuscitation (ECPR) was introduced in the 1960s to improve the efficacy and outcomes of cardiopulmonary resuscitation (CPR) when CPR fails [5]. Chen et al. [6] have used ECPR since 1994 and suggested that prolonged CPR rescue by extracorporeal membrane oxygenation (ECMO) might provide an acceptable survival rate and outcome in survivors, which was supported by subsequent studies [4, 7–10]. The International Liaison Committee on Resuscitation stated that ECPR might improve outcomes after CA compared with standard CPR in cases of cardiogenic shock and CA when there is an underlying circulatory disease amenable to immediate corrective intervention [11].

Despite significant risks, many institutions accumulated successful experiences with ECPR in adult patients [9, 12, 13]. The present study reviewed the 9-year experience of the Beijing Anzhen Hospital with ECPR performed in adult patients with post-cardiotomy CA. The aim was to observe outcomes during and after ECPR, and to identify factors that could affect the survival rate and weaning from ECMO. The hypothesis was that ECPR is effective in resuscitating patients with refractory CA after cardiac surgery.

## Methods

### Study population

This was a retrospective study of the 9-year experience (from January 2004 to May 2012) of the Beijing Anzhen Hospital with ECPR in adult patients with post-cardiotomy CA. CA was defined as the need for chest compressions or direct, open-chest cardiac massage [14, 15]. Inclusion criteria were: (1) cardiac origin of CA; (2) received ECMO after undergoing cardiac surgery in the hospital; (3) no sustained return of spontaneous circulation after at least 10–15 min; and (4) no obvious contraindication to ECPR including terminal malignancy, irreversible multi-organ failure, or severe neurologic injury. Exclusion criteria were: (1) patients who could not be weaned off cardiopulmonary bypass (CPB) after surgery owing to myocardial stunning and who were then shifted to ECMO; or (2) patients with progressive deterioration and who urgently needed ECMO support without cardiac massage or boluses of epinephrine.

As required by hospital policies, all cardiac surgery patients must sign an informed consent form with detailed explanations of the surgery as well as all optional procedures (e.g., ECPR) prior to the operation. This study was approved by the Capital Medical University in Beijing, China. All ECPR patients were identified from data collected at the Beijing Anzhen Hospital, and individual consent was waived by the committee because of the retrospective nature of the study. Data are available upon request addressed to the ethical committee of the Beijing Anzhen Hospital.

### ECMO team and organization

At the Beijing Anzhen Hospital, there is a dedicated ECMO rapid response team that is directly supervised by the Cardiac Intensive Care Center of the institution. This team consists of cardiac surgeons, intensive care unit (ICU) physicians, ECMO specialists and nursing staff. Per protocol, conventional CPR is managed by a fellow or cardiac surgeon, attending physician, ICU physicians, and nursing staff, all of whom provide assistance and consultation to the ECMO specialists. Those full-time ECMO specialists are not only responsible to help set up the ECMO circuit, but also responsible for ECMO care. The ECMO coordinator is on-call 24/7, and immediately available to return to the hospital to initiate ECPR.

### ECMO equipment and management

The ECMO cart includes cannulas, ECMO accessories, surgical instruments, suture materials, surgical drapes, and all necessary supplies. The ECMO system consists of a Quadrox-D hollow-fiber oxygenator with BIOLINE coating, a Rotaflow centrifugal pump (Maquet, Hirrlingen, Germany) with heparin-coated circuit tubing, a Sechrist oxygen/air blender, and a water heater/cooler (Sarns, Minneapolis, MN). Carmeda heparin-coated cannulas (Medtronic, Minneapolis, MN, USA) were used in all patients. Systemic heparinization was not needed on the day ECMO support was initiated; no hemorrhage, surgical bleeding, or oozing was noted due to heparin coating of the bioactive surface. The circuit was assembled and ready to be primed using acetated Ringer’s solution. All patients received venous-arterial (VA) ECMO for ECPR.

The femoral route was always preferred instead of the open sternotomy route for VA ECMO support because the wound of an open sternotomy increases the risk of infection and hemorrhage, and access via an open sternotomy also makes nursing care more difficult. If the femoral vascular status was poor, transthoracic cannulation was used. For femoral cannulation, the modified open Seldinger method was used [16]. The femoral vessels were dissected, and the cannulas were inserted with a guide wire under direct vision. This method was particularly useful during CPR when the femoral pulse was not palpable.

Cardiac massage was temporarily stopped for a few seconds when the vessel was punctured and the guide wire was introduced. Purse-string sutures were placed around the cannula to prevent hemorrhage. For distal extremity perfusion, an anterograde reperfusion catheter was inserted into the distal femoral artery.

When ECPR was initiated, the ECMO flow was set to achieve adequate blood pressure (approximately 60 mmHg) with adjustments using vasopressors and reversal of metabolic acidosis. Mixed venous oxygenation saturation was maintained to >65 % and hematocrit to >30 %. A constant positive end-expiratory pressure was maintained to keep the alveoli open. Core body temperature was kept at 34–35 °C using the heater-cooler load in the ECMO circuit for at least 24 h for cerebral protection in post-resuscitation care [17]. Electrolytes were corrected to the normal range, while the blood glucose was maintained to <200 mg/dl. To address this, fentanyl was used to sedate the patients after return of spontaneous eye movement; and serum lactate levels were measured to confirm the reversal of anaerobic metabolism. Low-dose heparin was infused to keep the activated clotting time between 180 and 220 s. High-dose catecholamine was slowly tapered down. If heart contractility improved based on echocardiography, if lung function was adequate, and if the shock status was reversed, an attempt was made to wean the patient from ECMO [18].

### Data collection

Demographic data including age, gender, height, weight, primary diagnosis, and pre-ECMO co-morbidities were recorded. The medical record consisted of CPR duration, ECMO duration, and ECMO-related complications. ECMO duration was defined as the time of ECMO flow initiation to the time of ECMO flow discontinuation [19]. Laboratory data included arterial blood gas (ABG) obtained pre-ECPR and 24 h after initiating ECPR, coagulation tests, and biochemical indices such as blood urea nitrogen (BUN), creatinine (Cr), aspartate aminotransferase (AST), alanine aminotransferase (ALT), total bilirubin (TBIL), direct bilirubin (DBIL), MB fraction of creatinine kinase (CK-MB), and lactic acid (LAC).

The clinical outcomes in the survival and non-survival groups were examined, and the outcomes of weaning from ECMO were also compared.

### Statistical analysis

All data were analyzed using SPSS 18.0 (SPSS Inc., Chicago, IL, USA). The Chi-square test was used for categorical variables. Continuous variables were tested for normality using the Kolmogorov–Smirnov test. The Wilcoxon rank-sum test was used for non-normally distributed variables. Normally distributed continuous variables were compared using the Student’s *t* test. All tests were two-sided. *P* values <0.05 were considered to be statistically significant.

## Results

### Characteristics of the patients

**Fig. 1:**
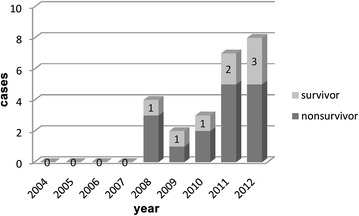
Yearly distribution of ECPR cases

Between January 2004 and May 2012, 150 patients underwent cardiac surgery and received ECMO. Twenty-four adult patients (19 males and 5 females) suffered from refractory CA and received ECPR. Mean age was 59.3 ± 11.9 years (range 23–76). Most patients were male (*n* = 19, 79.2 %). Of these 24 patients, 16 (66.7 %) were weaned from ECMO, 8 (33.3 %) survived to hospital discharge, and 16 (66.7 %) patients died. Diagnosis (*P* = 0.53) and ECPR location (*P* = 0.77) were not different between survivors and non-survivors (Table 1). Figure 1 shows the yearly distribution of ECPR cases and the survival-to-discharge ratio during the review period.

**Table 1:** Clinical characteristics

Variable	Survivors (*n* = 8)	Non-survivors (*n* = 16)	*P* values
Cardiac disease, *n* (%)			
CAD	8 (100.0)	12 (75.0)	0.530
AR	0	2 (12.5)	
VSD + PH	0	2 (12.5)	
ECPR location, *n* (%)			
Operation room	3 (37.5)	6 (37.5)	0.770
ICU	5 (62.5)	9 (56.2)	
Ward	0	1 (6.3)	

*CAD* coronary artery disease, *AR* aortic regurgitation, *VSD* ventricular septal defect, *PH* pulmonary hypertension, *CPR* cardiopulmonary resuscitation, *ECPR* extracorporeal cardiopulmonary resuscitation, *ICU* intensive care unit

### Characteristics of ECMO

The median duration of CPR from CA to the start of ECMO was 36.0 min (range 14–115). Mean duration of ECMO was 115.23 ± 70.17 h. Mean hospital stay was 25 days. ECPR was performed in 23 of the 24 patients using the femoral route. The open sternotomy route had to be used in one patient because of severe plaques in the femoral arteries; he was successfully weaned off ECMO, but died of multiple system organ failure (MSOF).

### Comparison between survivors and non-survivors

Tables 2 and 3 show the comparison between the survivors at discharge and the non-survivors. The demographic and biochemical data were not different between survivors at discharge and non-survivors before, during, and after ECMO. In addition, the duration of CPR and ECMO were similar in the two groups. Mean hospital stay for the non-survivors was 17.7 ± 9.5 days including 12.5 ± 8.1 days in the ICU. Mean hospital stay for the survivors was 41.0 ± 12.2 days including 8.7 ± 5.5 days in the ICU (Table 3).

**Table 2:** Patients’ characteristics before ECMO according to survival

	Survivors (*n* = 8)	Non-survivors (*n* = 16)	*P* values
Age (years)	56.6 ± 10.7	64.5 ± 8.0	0.125
Gender (male/female)	7/1	12/4	0.631
Hyperlipidemia	5 (52.5)	8 (50.0)	0.679
DM	2 (25.0)	4 (25.0)	1.000
Hypertension	2 (25.0)	8 (50.0)	0.388
Previous cerebral infarction	0	3 (18.8)	0.526
CPR duration (min)	30.00 (152.00)	43.00 (107.00)	0.976
Hypothermia	2 (25.0)	5 (31.3)	1.000
Pre-ECMO IABP	8 (100.0)	13 (81.3)	0.439
Distal perfusion	5 (52.5)	13 (81.3)	0.362
EF (%)	51.50 ± 14.82	52.00 ± 14.38	0.937
pH	7.35 ± 0.12	7.35 ± 0.13	0.909
PO2 (mmHg)	268.05 ± 117.89	174.89 ± 113.60	0.075
PCO2 (mmHg)	36.03 ± 5.97	39.44 ± 11.36	0.437
HCO_3_ˉ (mmol/L)	20.65 ± 4.17	21.69 ± 6.53	0.686
Base excess (mmol/L)	−4.75 (4.93)	−4.70 (7.78)	0.624
TBIL (μmol/L)	11.03 (4.62)	11.16 (15.63)	0.580
DBIL (μmol/L)	3.55 (1.54)	3.59 (3.29)	0.326

Results are presented as *n* (%), mean ± SD or median (interqartile range)

*ECMO* extracorporeal membrane oxygenation, *DM* diabetes mellitus, *IABP* intra-aortic balloon pump, *EF* ejection fraction, *BUN* blood urea nitrogen, *TBIL* total bilirubin, *DBIL* direct bilirubin

**Table 3:** Patients’ characteristics during and after ECMO according to survival

	Survivors (*n* = 8)	Non-survivors (*n* = 16)	*P* values
ECMO duration (h)	115.50 (22.00)	109.72 (69.62)	0.602
Kept pre-ECMO IABP	5 (62.5)	10 (62.5)	1.000
New intra-ECMO IABP	0	1 (6.3)	1.000
Cardiac tamponade	0	4 (25.0)	0.262
Re-CPR	2 (25.0)	5 (31.3)	1.000
Bacterial infection	5 (52.5)	6 (37.5)	0.390
Pneumorrhagia	0	3 (18.8)	0.526
Gastrointestinal bleeding	2 (25.0)	3 (18.8)	1.000
Urinary bleeding	0	2 (12.5)	0.536
Hemoglobinuria	1 (12.5)	3 (18.8)	1.000
Creatinine ≥3 μmol/L	1 (12.5)	8 (50.0)	0.178
Hemodialysis	1 (12.5)	6 (37.5)	0.352
Distal ischemia	1 (12.5)	1 (6.3)	1.000
Infection	5 (52.5)	6 (37.5)	0.390
MSOF	0	12 (75.0)	0.001
Brain death	0	2 (12.5)	0.536
Ventilation duration (h)	165.00 (304.00)	146.94 (91.357)	0.540
ICU duration (days)	12.50 (8.10)	8.77 (5.46)	0.158
Initiation flow (ml/min/kg)	47.20 ± 13.62	50.01 ± 11.60	0.603
Highest Lactate (mmol/L)	15.25 (4.00)	12.74 (4.55)	0.444
Highest creatinine (μmol/L)	77.25 (24.79)	85.00 (23.60)	0.581
Peak BUN (nmol/L)	18.04 ± 6.18	30.39 ± 25.78	0.086

Results are presented as *n* (%), mean ± SD or median (interquartile range)

*IABP* intra-aortic balloon pump, *ECMO* extracorporeal membrane oxygenation, *CPR* cardiopulmonary resuscitation, *MSOF* multiple system organ failure, *BUN* blood urea nitrogen

Among the non-survivors, 12 of 16 (75 %) patients died from MSOF. The incidence of MSOF was significantly higher in non-survivors (*P* = 0.001) (Table 3). One patient died from massive hemorrhage, despite undergoing a second operation. One patient had a left ventricular assistance device implanted concurrently, and could not be weaned from ECMO. Eight non-survivors had severe neurologic impairments. In addition, one survivor had neurologic complications (right lower extremity paralysis), and he was transferred to another specialized hospital for further treatments.

### Survival after weaning from ECMO

Sixteen patients who were weaned from ECMO were further divided into two groups: survivors at discharge (*n* = 8) and non-survivors after weaning from ECMO (*n* = 8). Some patients’ characteristics were different between survivors at discharge and non-survivors: duration from the end of surgery to CPR (median 4.5 vs. 95 min; *P* = 0.024), peak creatinine levels (158.91 ± 113.48 vs. 309.11 ± 139.27 μmol/L; *P* = 0.033), and peak BUN levels (median 18.04 vs. 29.09 mmol/L; *P* = 0.046) (Table 3).

### Comparison between non-survivors during ECMO and non-survivors after weaning ECMO

Non-survivors were divided into two groups (non-survivors of weaning ECMO and non-survivors during ECMO). Compared with patients who died during ECMO, patients who died after weaning showed a longer time between surgery and CPR [median 95.0 (248.0) vs. 2.5 (93.0) h, *P* = 0.034], higher pre-ECPR TBIL levels [median, 15.80 (30.30) vs. 8.83 (4.73) µmol/L, *P* = 0.011], higher frequency of consciousness during ECMO (87.5 vs. 12.5 %, *P* = 0.010), shorter ECMO duration [median, 111.4 (41.1) vs. 127.5 (128.0) h, *P* = 0.012], and lower peak CKMB levels [median 176 (96) vs. 283 (405) U/L, *P* = 0.035] (Table 4).

**Table 4:** Comparison between non-survivors after weaning from ECMO and non-survivors during ECMO

	Non-survivors after weaning from ECMO (*n* = 8)	Non-survivors during ECMO (*n* = 8)	*P* values
Pre-ECMO			
Age (years)	59.8 ± 17.7	61.5 ± 4.5	0.790
Gender (male/female)	4/4	8/0	0.077
Duration from operation to CPR (h)	95.00 (248.00)	2.50 (93.00)	0.034*
Pre-ECPR TBIL (μmol/L)	15.80 (30.30)	8.83 (4.73)	0.011*
Intra-ECMO			
Awake	7 (87.5)	1 (12.5)	0.010*
ECMO duration (h)	111.38 (41.08)	127.50 (128.00)	0.012*
Peak CKMB (U/L)	176.02 (95.88)	282.50 (405.00)	0.035*
Peak lactate (mmol/L)	12.79 (6.35)	20.00 (4.60)	0.067
Peak creatinine (μmol/L)	268.05 ± 117.89	174.89 ± 113.60	0.051
Lactate 24 h after ECPR (mmol/L)	4.65 ± 3.90	6.10 (10.10)	0.074
Bleeding at intubation site	0	4 (50.0)	0.077

Results are presented as *n* (%), mean ± SD or median (interquartile range)

*ECPR* extracorporeal cardiopulmonary resuscitation, *CKMB* creatinekinase-MB, *TBIL* total bilirubin

* *P* < 0.05

### Complications

Eleven patients (45.8 %) developed bacterial infections. Clinical complications of ECPR such as cardiac tamponade, brain lesion, pneumorrhagia, urinary tract bleeding, and persistent hemorrhage only occurred in the non-survivors. Mechanical complications such as thrombosis in the pump head or distributed within the extracorporeal circulation also occurred only in the non-survivor group.

## Discussion

The objective of the present study was to examine the clinical outcomes of ECPR in adult patients with post-cardiotomy refractory CA. Results showed that among 24 adult patients who received ECPR support for post-cardiotomy refractory CA, the mean ECMO duration was 115.23 ± 70.17 h. The cardiac surgery procedures included coronary artery bypass grafting (83.3 %), valvular surgery alone (8.3 %), and correction of congenital heart defects (8.3 %). Twenty-one patients received ECPR after intra-aortic balloon pump (IABP) and three patients received ECPR directly. The main cause of mortality was multiple system organ failure (50.0 %). Approximately one-half of non-survivors had severe neurologic impairments. Among 16 patients who were weaned from ECMO, eight patients were alive at discharge.

ECPR provides a period of stability for the resolution of the underlying problems that led to refractory CA in the first place [20]. Because of the cost, complexity of the technique, and required resources, ECPR is not offered in all centers. However, ECPR is recommended for CA that is refractory to initial resuscitation attempts if the condition leading to CA is reversible or amenable to heart transplantation, if excellent conventional CPR has been performed after no more than a few minutes of CA, and if the institution is able to rapidly perform ECMO [21].

In the present study, CABG (83.3 %) was the most common surgical procedure that was performed in patients who developed CA and required ECPR, which is in agreement with a previous report [22]. It is unclear whether or not patients with coronary artery diseases or other cardiac diseases are more sensitive to reperfusion injury of ischemic tissue, and whether or not they are more susceptible to the possibility of myocardial injury following ECPR [17]. Further studies are necessary to assess this point.

In the study by Lan et al. [22], the survival rate was 30.1 %, while the study by Flecher et al. [23] reported survival of 41–45 %. According to the Extracorporeal Life Support Organization (ELSO) registry, the survival rate of adult patients receiving ECPR for CA was 28 % between 1990 and 2012 [24]. Although the survival rate in the present study was higher, it was comparable to previous studies [9, 25]. However, the ELSO registry includes all patients receiving ECPR, while the present study included patients who underwent cardiac surgery only. Another study in a population of patients that was similar to the present study showed a survival of only 15 % [26]. As in the study by Lan et al. [22], gender did not affect survival at discharge.

In the present study, the duration of CPR and ECMO were also similar to those of Huang et al. [19], and did not differ significantly between survivors and non-survivors. In our hospital, the ECMO preparation room is next to the ICU and operating room; therefore, ECMO preparation and initiation are very timely if CA occurs. Nine patients (37.5 %) suffered from CA at the end of the surgery, and there was no need to move them before initiating ECPR. Fourteen patients (58.3 %) suffered from CA in the ICU. In the present study, set up time for ECPR (from notification of the ECMO team to running the ECMO pump) was approximately 30 min. According to recent reports on CPR, the sooner ECPR is initiated, the earlier systemic perfusion is improved, and the higher the survival rate [19, 27]. In the present study, there was a trend for higher mortality rates with increasing CPR duration.

An IABP is the first option due to its relative non-invasiveness and low cost compared with ECMO. In contrast, ECMO is the better choice for CA not amenable to IABP support alone. Twenty-one patients (87.5 %) were New York Heart Association (NYHA) class III or IV prior to surgery, and they had been using a preoperative or intraoperative IABP. Refractory CA eventually occurred in this population after surgery because of the inefficacy of IABP. For such patients, ECMO alone or in combination with IABP might be used as soon as possible, but further studies are necessary to evaluate this approach.

According to several published studies, sepsis with MSOF has been associated with poor outcomes [22]. However, there was no difference in the frequency of infections in the present study. All patients of the non-survivor group with bacterial infections eventually developed MSOF. Control of infections is still a major challenge in the ICU. In the present study, MSOF was the primary cause of death. Patients who do not receive chest compressions usually undergo a medical resuscitation using potent drugs that maintain the cardiopulmonary function at the expense of other organ systems such as the kidneys [28]. In the present study, survivors at discharge had better creatinine and peak BUN levels than non-survivors, and a larger proportion of non-survivors were undergoing hemodialysis. As in the study by Grist et al. [20], patients sustained severe kidney damage with a dismal outcome, despite hemodialysis, and still lacked adequate renal perfusion after ECMO.

A previous study reported that age >60 years, requirement of postoperative VA hemofiltration, peak TBIL >102.6 µmol/L, and a need for ECPR were independent risk factors for in-hospital mortality [29]. Another study reported that patients aged >65 years, pH <7.0, lactates >12 mmol/L, creatinine >200 µmol/L, or receiving ECMO under advanced life support had a bad prognosis [23]. However, these factors could not be validated in the present study, mostly because of the small sample size. Indeed, as a retrospective, non-randomized, observational study, the present study had a number of limitations. Although this study collected the neurological outcome data from all patients, detailed neurologic examinations and neurodevelopmental evaluations were not recorded, as was the case in other series [19, 20, 22]. Many variables only showed a trend toward a difference, maybe due to the small sample size, which also prevented subgroup analyses. A larger multicenter randomized study is required to correctly assess the benefits of ECPR in patients with refractory CA after cardiac surgery. In addition, the etiologies of CA after cardiac surgery were not collected in the present study.

Similar to Kelly et al. [15], we hypothesize that early ECPR and effective care are necessary to the effectiveness of ECMO for refractory CA after cardiac surgery. Given that medical and surgical complications may occur during any phase of care, we emphasize the importance of multidisciplinary collaboration between professionals involved in the care of these patients [30]. However, a recent meta-analysis reported that significant morbidities were associated with ECMO, and that its use should be carefully considered based on the risk–benefit ratio [31].

## Conclusions

ECPR can be successful to resuscitate adult patients following refractory CA after cardiac surgery. Because ECPR is an aggressive therapy, it is important to carefully select the patients for ECPR, and combine ECPR with IABP if necessary to improve the efficacy. Improvements in outcomes of patients undergoing ECPR also require a multidisciplinary care approach to protect organ function and limit organ injury before and during ECMO support. Educating ECMO staff and improving selection of appropriate patients for ECPR as early as possible during CPR should contribute to an increased survival rate.

## Abbreviations

ABG: arterial blood gas
ALT: alanine aminotransferase
AST: aspartate aminotransferase
BUN: blood urea nitrogen
CA: cardiac arrest
CK-MB: MB fraction of creatinine kinase
CPB: cardiopulmonary bypass
CPR: cardiopulmonary resuscitation
Cr: creatinine
DBIL: direct bilirubin
ECMO: extracorporeal membrane oxygenation
ECPR: extracorporeal cardiopulmonary resuscitation
IABP: intra-aortic balloon pump
ICU: intensive care unit
LAC: lactic acid
MSOF: multiple system organ failure
NYHA: New York Heart Association
TBIL: total bilirubin
